# Loosening Monitoring of the Threaded Pipe Connection Using Time Reversal Technique and Piezoceramic Transducers

**DOI:** 10.3390/s18072280

**Published:** 2018-07-14

**Authors:** Yabin Liang, Qian Feng, Dongsheng Li

**Affiliations:** 1 Hubei Key Laboratory of Earthquake Early Warning, Institute of Seismology, CEA, Wuhan 430071, China; yabinliang@eqhb.gov.cn; 2 Wuhan Institute of Earthquake Engineering Co. Ltd., Wuhan 430071, China; 3 Department of Civil Engineering, Shantou University, Shantou 515063, China; lids@stu.edu.cn

**Keywords:** lead zirconate titanate (PZT) transducer, loosening monitoring, threaded pipe connection, time reversal technique

## Abstract

Threaded pipe connections are commonly used in the oil and gas industry in particular to connect casting strings, drill pipe strings, production and transportation risers, and pipelines. As the most critical components in the entire chain, maintaining a sealed and secure connection while being subjected to environmental loads and pollution is very important and necessary to reduce potential leakage risk and guarantee the safety of the entire chain. In this paper, an effective approach using time reversal technique and lead zirconate titanate (PZT) transducer was developed to monitor the looseness of the threaded pipe connection. Two threaded pipeline segments connected with a metal coupling were assembled to simulate the threaded connection in the pipeline system. Two PZT patches were mounted on the surface of one pipeline segment and the pipe coupling, respectively. By loosening the threaded connection with different rotation angles, several looseness scenarios were experimentally investigated. For each looseness condition, the developed time reversal-based approach was performed and the corresponding response signal was acquired and analyzed. The experimental results demonstrate that the peak value of the focused signal detected by the PZT sensor decreases with the increase of the looseness degree. The entire test conducted from tightened connection to loosened connection was repeated eight times to validate the repeatability of the developed method and the consistency of the detection results. In addition, the reliability of the developed method was studied by involving high disturbances when the signal was measured. All the test results show that the developed method has a great potential to be employed in practical applications for monitoring the looseness condition of the threaded pipe connection, especially in an environment with severe noises and disturbances.

## 1. Introduction

With the advantages of flexibility for assembly and disassembly, excellent bearing capacity for large axial force, good interchangeability and reusability, threaded pipe connections are commonly used in the oil and gas industry in particular to connect casting strings, drill pipe strings, production and transportation risers, and pipelines [1,2,3]. As the most critical components in the entire chain, maintaining a sealed and secure connection while being subjected to environmental loads and pollution is very important and necessary to reduce potential leakage risk and guarantee the safety of the entire chain [4,5].

For pipeline projects, when a visual inspection is not possible in case of buried pipeline, the presence of a leak can be identified by a drop of the local pressure measured by the pressure sensors [6]. However, the reliability of this method is relatively poor due to the influence of the temperature difference along the pipeline. Therefore, some new solutions for monitoring and detection of the pipeline leakage were proposed by researchers in recent years [7,8,9]. For example, Zhou et al. [10] and Liang et al. [11] demonstrated that the leakage along the pipelines can be detected and located by monitoring the variation of the pipeline surrounding temperature using the distributed temperature sensing system (DTS) combined with optical fibers. Qu et al. [12] presented a support vector machine (SVM) based pipeline leakage detection and pre-warning system by employing the distributed optical fiber sensors, thus the abnormal events of the pipeline can be identified by analyzing the vibration signals caused by the defects of the pipeline. For threads failure of pipeline structures, He et al. [13] presented a non-contact detecting method for oil tube thread, in which, the optical techniques and image processing techniques were employed to measure the physical dimensions of the tube thread and give further analysis. Chen et al. [14] presented a new analytical method that can calculate the load distribution on the thread teeth in cylindrical pipe threaded connection by analyzing each male and female thread tooth from the connection on the basis of elastic mechanics.

On the other hand, due to the availability in different shapes, broadband response frequency, low price, and the ability of being employed as actuator and sensor simultaneously, piezoceramic Lead Zirconate Titanate (PZT) materials and PZT-based approaches have been widely recognized as one of the most promising techniques in the area of structural health monitoring (SHM) for engineering structures in recent years [15,16,17,18,19]. By combining with different defect diagnose algorithms, these piezoceramic transducers and related piezocermic-based techniques were developed to investigate the connection status of the structural connection components, such as the pin-connection [20,21,22], the bolted connection [23,24] and the cuplok connection [25]. For example, Hong et al. [26] proposed a dynamic cooperative identification method (DCIM) for pipe SHM with PZT-based transducers, and the performance of the proposed methodology for damage identification was successfully validated by an experimental investigation of a pipe structural model with various connector damage scenarios created by loosening connectors. All the research demonstrates that the piezoceramic-based methods have the great potential to achieve the damage detection and loosening monitoring for structural connection components.

As one of the most effective methods in the area of structural health monitoring, time reversal (TR) technique was first introduced by the modern acoustics community [27,28], and then was adopted to Lamb wave based NDT in order to compensate for the dispersion of Lamb waves and to detect defects in a pulse-echo mode [29,30]. The main interest of these studies was refocusing energy in the time and spatial domain by compensating for the dispersive characteristic of Lamb waves [31]. Hsu [32] applied finite element method to simulate the propagation of the guided wave through the defect on the elbow part in pipe, and the TR technique was employed to diagnose the multiple defects exist in the pipes. Zhang et al. [25] investigated the effectiveness of the TR technique to be used to analyze the transmitted signal between the PZT patches through the cuplok connection, and the experimental results proved that the peak value of the TR focused signal is capable of monitoring the tightness of cuplok connection. Similarly, Liang et al. [20] successfully applied the TR technique to monitor the load variation of the pin-connected structure, and the experimental results demonstrated that the TR technique has a good repeatability and anti-disturbance ability.

In addition, Hong et al. [33] developed an active monitoring method, which combined the TR technique with piezoceramic transducers, to help identify the looseness condition of the tapered threads connection. In his research, the inherent relationship between the contact area and tightness degree of tapered threads connection for the pipeline specimen was studied, and the performance of the approach was experimentally validated. However, some important factors, which may affect the results were not considered in his research. For example, (1) the influence of the relative distance variation between the PZT actuator and sensor was not considered when analyzing the propagation and attenuation of the stress wave energy, (2) the repeatability of the proposed approach and the consistency of the results were not investigated, (3) the reliability of the TR-based technique when subjected to high environmental disturbances was not considered. Therefore, monitoring the looseness condition of pipeline tapered threads connection using TR technique still remains many problems and requires further study.

To solve these problems, an effective approach using a combination of TR technique and piezoceramic transducers was developed in this research to quantitatively monitor the looseness condition of the threaded connection in pipeline system. In addition to studying the feasibility of the proposed method, this research also conducted the following studies which were never reported in the literature:

(1) The combined effect of the contact area change of the screwed interface and the relative distance variation between the PZT actuator and sensor to the results.
(2) The repeatability of the developed TR-based approach and the consistency of the results.
(3) The anti-disturbance performance of the developed approach.

In the study, two threaded pipeline segments connected with a metal coupling were assembled to simulate the threaded connection in the pipeline system, and two PZT patches were surface bonded on one pipeline segment and the pipe coupling, respectively. Then, several different looseness scenarios were artificially introduced and experimentally investigated by loosening the threaded connection of the prepared specimen with different rotation angle. For each looseness condition, the developed TR-based approach was performed and the corresponding response signal was acquired and analyzed. The peak value of the focused signal detected by the PZT sensor was selected and analyzed as the looseness-monitoring index to identify the looseness and quantify its severity. The entire test conducted from tightened connection to loosened connection was repeated eight times to validate the repeatability of the developed method and the consistency of the detection results. In addition, the reliability of the developed method was studied by involving high disturbances when signal was measured. All the test results show that the developed method has a great potential to be employed in practical application for monitoring the looseness condition of the threaded pipe connection, especially in an environment with severe noise and disturbances.

## 2. Detection Principles

### 2.1. Threaded Pipe Connection

**Figure 1:**
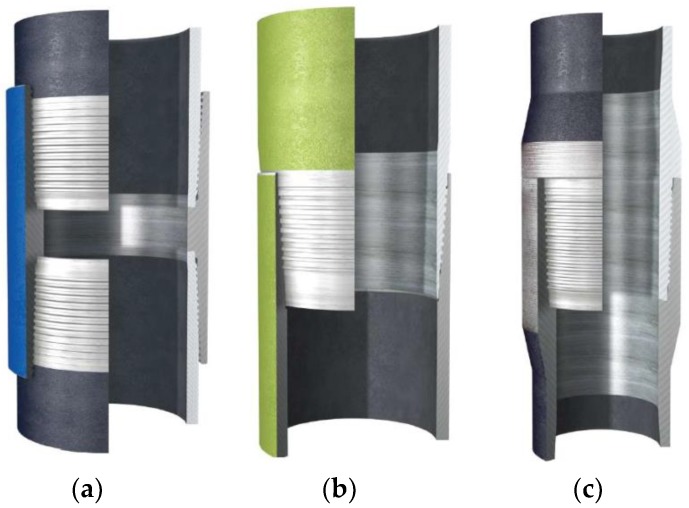
(**a**) Threaded and coupled, (**b**) integral flush, (**c**) integral upset connection [35].

The threaded pipe connections consist of pipes and coupling part, called respectively pin and box, can be classified into three different types, as shown in Figure 1. The first type is called Threaded and Coupled (T&C) since the pipes carry male threads at both ends and a separate coupling part is used to connect them. These connections are commonly used in casting tubing and riser applications. The second type is called Integral Flush type, because no separate coupling part is used and the pipes have a male and female part of the connection at either end. When the connection is produced in the pipe material without any local increase of inside or outside diameter, it is called a flush connection. However, when the connection is fabricated in a part with a thicker wall than the rest of the pipe, it is called an Upset connection [1,2,3]. Integral flush connections are used in casing pipes while integral upset connections are more commonly used in tubing and drill pipes [34]. In this paper, the pipeline specimen with the first type of connection was studied to validate the effectiveness of the developed TR-based loosening monitoring method with piezoceramic transducers.

### 2.2. Stress Wave Propagation through Threaded Pipe Connection

**Figure 2:**
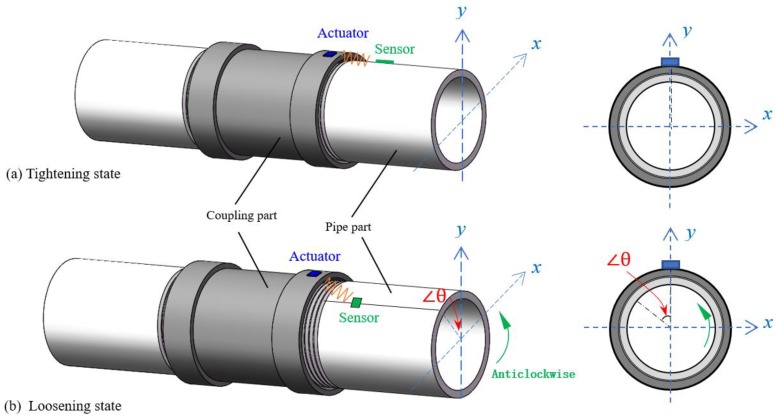
The schematic of the stress propagation between the lead zirconate titanate (PZT) actuator and PZT sensor on threaded pipe connection.

As shown in Figure 2a, when the threaded connection is under the tightening state at the beginning, one PZT patch as an actuator is bonded on the outside surface of the coupling part, and the other PZT patch as a sensor is bonded on the pipe surface near the coupling part. Subsequently, a desired stress wave excitation is generated by the actuator and then propagated through the contact interface of the coupling and pipe segment of the connection. The wave response signal was finally received by the PZT sensor. As shown in Figure 2b, assuming the pipe is loosened from the coupling part, more propagation wave energy from the actuator to the sensor will attenuate due to two main reasons, i.e., the change of the threaded interface and the change of the propagation distance.

(a) The threaded interface change

**Figure 3:**
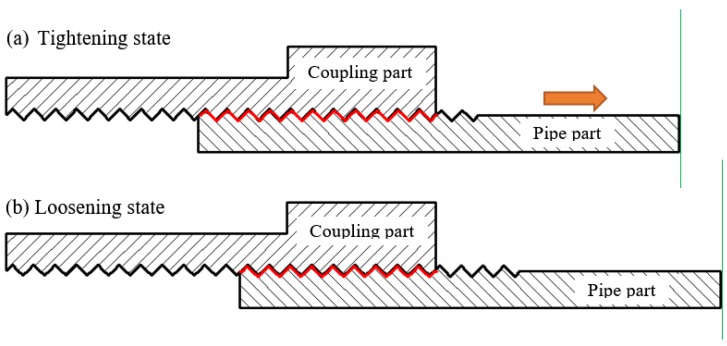
The reduction of the contact area for the connection interface caused by pipe looseness.

When pipe looseness occurs, the area of the contact interface between the pipe and the coupling reduces, as shown in Figure 3. Previous related researches have demonstrated that the increase of the contact area between the structural interfaces will help propagate the stress wave. In addition, a monotonic relationship can be observed between the contact area and the magnitude of the received signal response [36,37]. Therefore, with the decrease of the contact area of the connection when pipe looseness occurs and develops, the received stress wave energy will correspondingly decrease.

(b) The propagation distance change

As shown in Figure 2b, assuming the pipe is loosened from the coupling by anticlockwise rotating the pipe part with a certain rotation angle, thus the location of the PZT sensor, which was surface bonded on the pipe part, changes because of the looseness, and finally induce the change of the relative distance between the PZT actuator and sensor.

**Figure 4:**
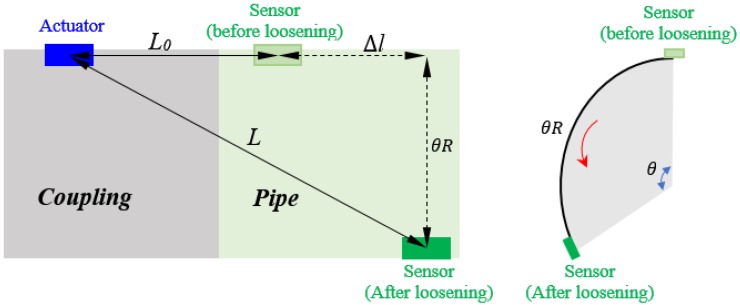
The change of the relative distance between the PZT actuator and sensor because of the looseness with a rotation angle θ.

In the study, assuming the initial distance between the PZT actuator and PZT sensor is *L*_0_ and the external diameter of the pipe part is *R*, as shown in Figure 4, when the pipe part was rotated anticlockwise with an angle θ, the distance between these two PZT patches increase (denoted as *L*) and can be easily calculated by employing the Pythagorean proposition algorithm. In other words, when the looseness occurs, the updated relative distance *L* has a positive correlation with the rotation angle θ with a range from 0 to π. A few researches done by other researchers have theoretically and experimentally verified that the attenuation of the stress wave energy increases with the increase of the wave propagation distance [38,39,40]. Therefore, in this study, the increase of the propagation distance caused by the looseness of connection will increase the stress wave energy attenuation and reduce the energy magnitude of the received signal response.

Based on above analysis, it can be summarized that when the looseness occurs, changes of the stress wave energy computed from the received signal of the PZT sensor (denoted as Δ*E*) can be contributed by two parts, one is the energy change caused by the contact area variation of the connection, denoted as $\Delta E_{\Delta S}$, and the other one is the relative distance change between the PZT sensor and actuator, denoted as $\Delta E_{\Delta L}$. The relationship can be expressed as

(1) $$\Delta E=\Delta E_{\Delta S}+\Delta E_{\Delta L}\;$$

In addition, it has been described above that the looseness occurrence and development can be considered as the increase of the relative rotation angle θ between the pipe and coupling part. Both the $\Delta E_{\Delta S}$ and $\Delta E_{\Delta L}$ have positive correlation with the increase of rotation angle θ. Therefore, a conclusion can be drawn that the occurrence and development of the pipe looseness for the threaded pipe connection can be potentially detected by monitoring the energy attenuation of the desired stress wave propagated through the contact interface of the connection.

### 2.3. PZT-Based Time Reversal Technique

In recent years, time reversal technique has attracted increasing attentions because of the unique advantage to compensate the dispersion of Lamb waves and to improve the signal-to-noise ratio of the propagating waves [29,41,42,43]. By combining with the piezoceramic transducers, this technique has also been commonly applied in the area of structural health monitoring and damage diagnose [44,45,46,47]. In the study, the time reversal technique was employed to detect and monitor the pipe looseness of the threaded pipe connection.

**Figure 5:**
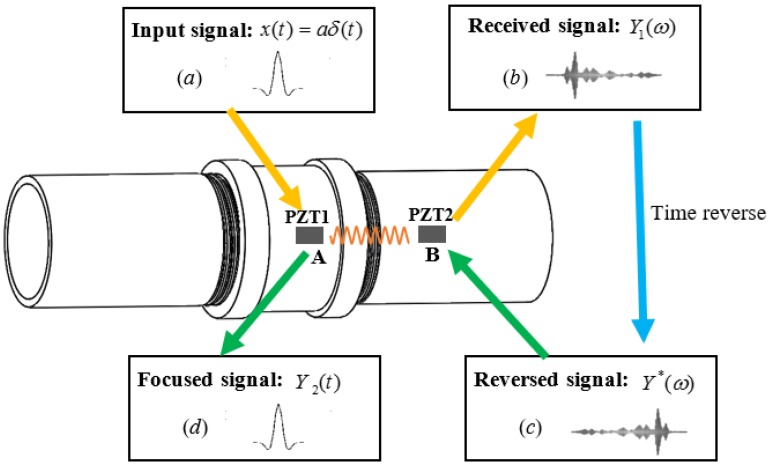
Theoretical procedure of time reversal technique.

According to conventional time reversal acoustics, an input signal can be focused at an excitation point if an output signal recorded at another points is reversed in the time domain and emitted back to the original source point [48]. In this study, assuming a pulse input signal *x*(*t*) was first generated by the PZT patch at A point and regarded as the excitation signal, then the acoustic field produced by this excitation signal propagated according to the medium characteristics, attenuation, velocity and dispersion, and finally another PZT patch received its wave response at B point, as shown in Figure 5. Therefore, the received signal *y*_1_(*t*) at B point due to the excitation signal *x*(*t*) at A point can be represented by

(2) $$y_{1}(t)=x(t)⊗g(t)\;$$

where *g*(*t*) is the structural transfer function for the given signal propagation patch, and ⊗ denotes the convolution operation. Then, transferring above equation from time domain to frequency domain, it becomes

(3) $$Y_{1}(\omega )=X(\omega )G(\omega )\;$$

where *Y*_1_(ω), *X*(ω) and *G*(ω) are the Fourier transform expressions of the received signal *y*_1_(*t*), the input signal *x*(*t*) and the transfer function *g*(*t*), respectively, when respect to the angular frequency ω. In Fourier space, time reversal of the received signal at B point is defined as

(4) $$Y(\omega )\overset{TR}{⟶}Y^{*}(\omega )\;$$

where * denotes the complex conjugate of the function. Therefore, the time reversal signal at B point can be represented by

(5) $$Y_{TR}(\omega )=X^{*}(\omega )G^{*}(\omega )\;$$

According to the related research [49,50], time reversal of the stress wave is based on the spatial reciprocity of stress wave propagation in a medium, which means the position of a source and receiver can be interchanged without altering the resulting field. Thus, the reversal signal *Y_TR_*(ω) is then resent from B point to A point, resulting in

(6) $$Y_{2}(\omega )=Y_{TR}(\omega )G(\omega )=X^{*}(\omega )G^{*}(\omega )G(\omega )\;$$

For most input signals, such as the Gaussian pulse, sinusoidal signals and square signals, they are all time reversal symmetric, i.e., *x*(−*t*) = *x*(*t*). Thus, when transferring into frequency domain, the input signal can also be represented by X*(ω) = *X*(ω). Therefore, Equation (6) can be rewritten as

(7) $$Y_{2}(\omega )=X(\omega )G^{*}(\omega )G(\omega )=X(\omega ){\left|G(\omega )\right|}^{2}\;$$

then transforming the signal back into the time domain, it becomes

(8) $$Y_{2}(t)=\frac{1}{2\pi }\int_{-\infty }^{\infty }X(\omega ){\left|G(\omega )\right|}^{2}e^{i\omega t}d\omega \;$$

If ${\left|G\left(\omega \right)\right|}^{2}$ is independent of ω, the signal at the end of the time reversal process is directly related to the original excitation signal and can be expressed as

(9) $$Y_{2}(t)=C\;x(t)\;$$

where *C* =$\;{\left|G\left(\omega \right)\right|}^{2}/2\pi$. More specifically, considering that the discrete-time input can be expressed as *x*(*n*) = *a*δ(*n*), where δ(*n*) denotes a unit pulse and a is the amplitude of input signal, then when *n* = 0, the focused signal *Y*_2_(*n*) has the maximum value, i.e., *Y*_2_(0) = *aC*.

Based on above theoretical derivation and analysis, it can be demonstrated that when the input signal is constant, the peak value of the focused signal is only related with the stress wave transfer function, which was determined by the medium characteristics for the test structures. In addition, the equations also reveal that the focused signal is uncorrelated to noise, and the peak value *Y*_2_(0) will not be affected by noise and disturbance. This result predicts that the time reversal technique is insensitive to the noise and has a potential to be used in a low Signal-Noise Ratio (SNR) environment.

## 3. Experimental Setup and Procedures

### 3.1. Specimen Preparation and Sensor Location

**Figure 6:**
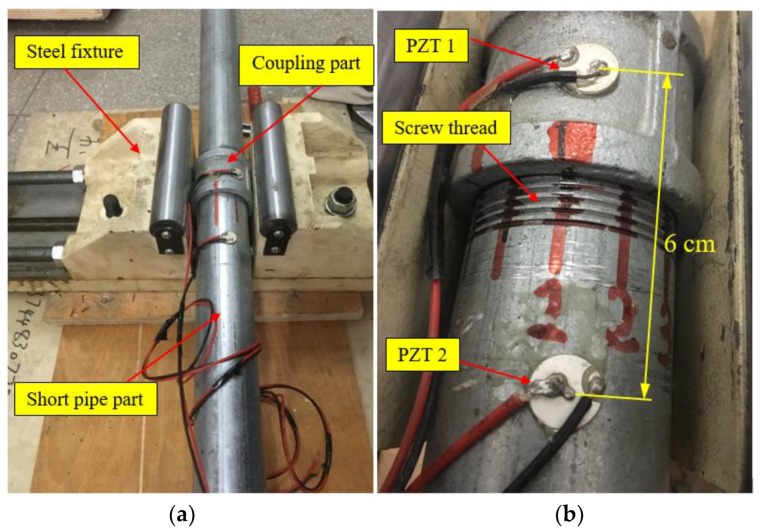
The test specimen of threaded pipe connection and the PZT sensors. (**a**) The test specimen; (**b**) The location of PZT sensors.

In order to validate the effectiveness of the developed method, an experimental investigation was conducted in this study. At first, a steel threaded pipe connection specimen with the Threaded and Coupled connection was prepared in the laboratory, as shown in Figure 6a. Two pipe segments and one coupling part were assembled and then fixed on the ground by a steel fixture. In the experiment, two PZT-5H patches were bonded on outside surface of the pipe part and coupling part of the connection, respectively, and the relative distance between them was about 6 cm, as shown in Figure 6b. The detail geometrical and material parameters of the test specimen and the PZT patches can be found in Table 1.

### 3.2. The Excitation Signal

Following the theoretical derivation of time reversal technique [40,50], the Gaussian-modulated sine signal was adopted in this study as a pulse waveform, which can be expressed by a formulation as,

(10) $$x(t)=Ae^{-k{\left(t-d\right)}^{2}}\cos\left(2\pi f_{c}(t-d)\right)\;$$

where

(11) $$k=\frac{5\pi^{2}b^{2}f_{c}^{2}}{q\ln(10)}$$

in which, *x*(*t*) is the generated excitation impulse signal at time point *t*, and *A*, *b*, *q*, *f_c_*, *d* denote the amplitude, the normalized bandwidth, the attenuation, the center frequency and the delay for the signal, respectively.

**Figure 7:**
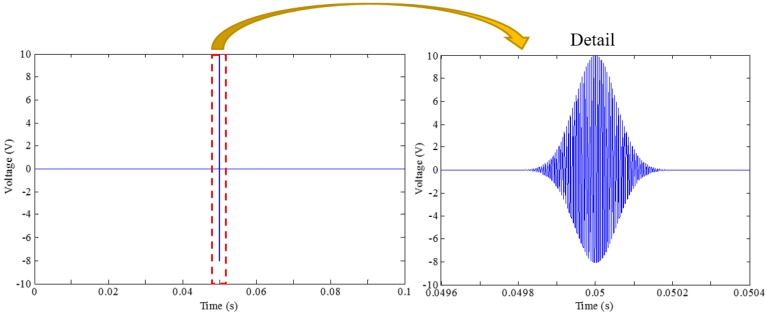
The pulse signal.

At the beginning, a swept frequency test with a frequency range of 100 Hz to 500 kHz, was pre-conducted between the PZT actuator to the sensor. The corresponding response signal presents an energy concentration near the frequency of 200 kHz, which was then selected as the center frequency of the impulse signal. The amplitude of 10 V for the pulse waveform was chosen according to the maximum specification of the data acquisition system channel output. Other detailed parameters of the excitation signal can also be founded in Table 2, and the generated pulse signal is plotted in Figure 7.

### 3.3. Instrumental Setup and Test Procedure

For threaded pipe connection, the torque was always employed as an important evaluation parameter to determine the tightness degree of connection during installation. However, research found that a same torque may correspond to different contact areas in various meshing processes of the threads, and it is unacceptable as monitoring variable to afford the research uniqueness to monitor the tightness of the threaded connection [33]. In this study, to figure out this relationship correctly, the relative rotation angle between the coupling and the pipe was introduced as a monitoring variable to indicate the changes of the contact area of the threaded connection when the looseness occurs and develops.

**Figure 8:**
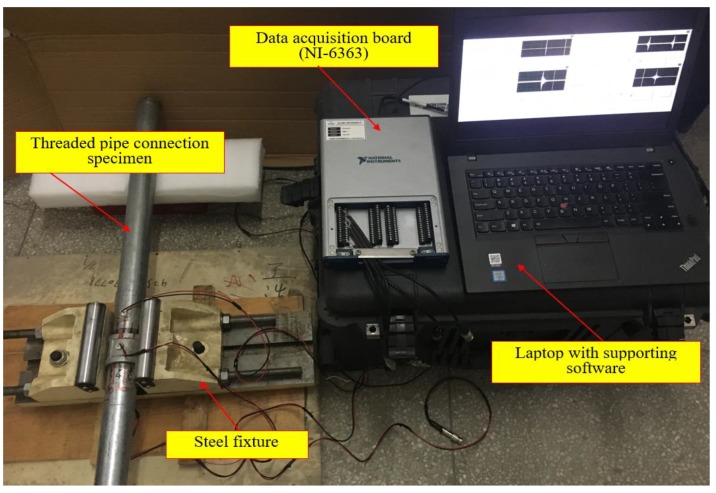
The instrumentation setup.

As shown in Figure 8, the coupling part of the specimen was fixed by a steel fixture. Thus, the pipe looseness of the connection could be artificially introduced by rotating the pipe segment with a pre-determined angle along the loosening direction, i.e., anticlockwise. The instrumentation setup includes the steel threaded pipe connection specimen, a NI-DAQ data acquisition board (NI-6363) and a laptop installed the supporting software.

As for the chosen specimen, its threaded connection would get really tight after the contact region outnumbers 5 circles by rotating the pipe segment, therefore the initial tightening state for the connection in the experiment was fixed at 5.5 circle, where the threaded connection was considered tight enough. In this situation, two PZT patches were surface bonded on the coupling and one of the pipe segments of the connection specimen, respectively, and located in a straight line along the length of the pipe specimen, as shown in Figure 6b.

In the practical application of threaded connections for pipeline system, monitoring its early disease and slight looseness is much more meaningful and necessary to reduce the leakage risk and guarantee the safety of the structures, and very loose states were unnecessary to be considered. In this experiment, assuming the rotation angle between the coupling and the pipe segment is zero at the initial tightened status, then seven slight looseness scenarios with different severities were artificially introduced and experimentally investigated by rotating the pipe segment with different rotation angles from 0 to 180° (1/2 circle) with a step increase of 30° (1/12 circle).

For each looseness status, an impulse signal was first generated and sent out as an output via the D/A interface of the NI data acquisition card, and then sent to PZT1, which was surface bonded on the coupling part. Upon excitation, PZT1 generated a stress wave that propagated through the threaded contact interface of the pipe and the coupling and was finally received by the surface bonded PZT2 on the pipe part. Then, the received signal at PZT2 was reversed in the time domain and subsequently resent from PZT2 back to PZT1. Finally, the received stress wave signal at PZT1 was transformed into electrical signal, which was acquired by the computer via A/D interface of the NI data acquisition card. In the experiment, both the output signal and the received signal were generated or acquired with a sampling frequency of 1 MHz and a duration of 0.1 s, which was sufficient to prevent or minimize the aliasing effects.

For the test specimen, upon excitation, the generated elastic waves propagate through the threaded pipe connection interface, interrogate the loosening severity of the connection, and thus provide information on the actual operation conditions of the connection. Therefore, the occurrence and development of the looseness for the threaded pipe connection can be detected and monitored by analyzing the stress wave propagation and attenuation.

## 4. Experimental Results and Analysis

### 4.1. The Relationship between the Stress Wave Transmission and Loosening Severities of the Connection

**Figure 9:**
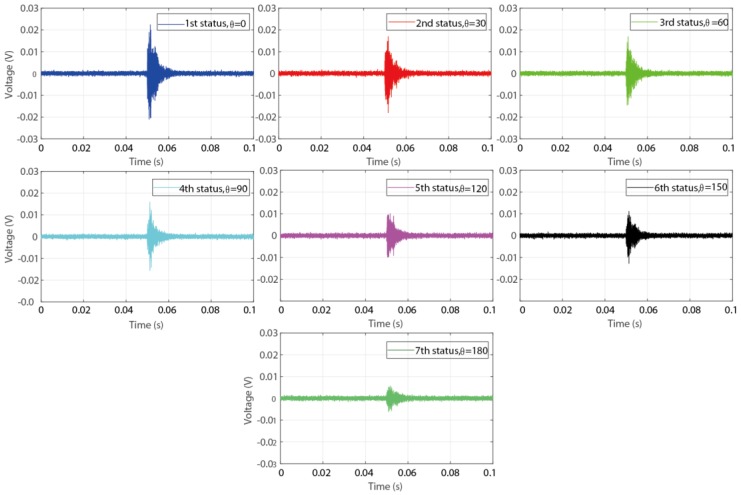
The wave response signal received by PZT2 in the first step.

**Figure 10:**
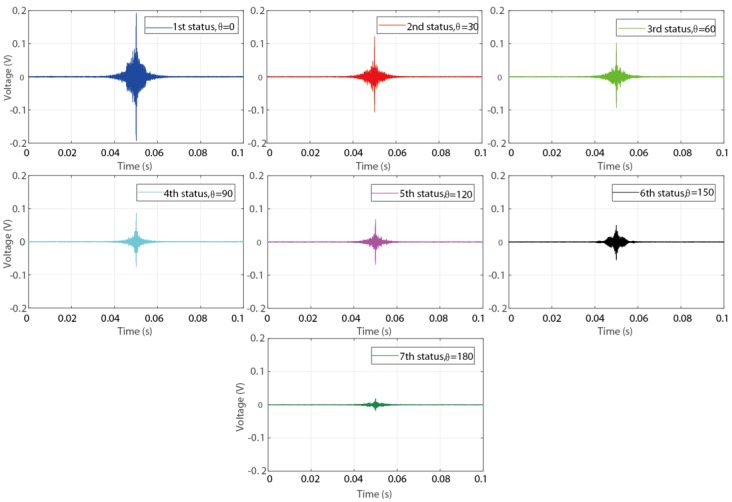
The focused signal received by PZT1 in the last step.

Based on above description, totally three steps were conducted during the detection procedure for the experimental investigation using time reversal technique. At first, an impulse sine waveform as the excitation signal was sent from PZT1, which was surface bonded on the coupling part, then the generated stress wave propagated through the contact interface of the connection, and was finally received by PZT2, which was surface bonded on the pipe part. Subsequently, a reverse operation was conducted for the received signal in the first step by reversing it in the time domain. Thirdly, the reversed signal was re-submitted by PZT2 as a new excitation signal, and the excited stress wave propagated following the opposite path in the first step. Finally, the wave response signal received by PZT1 was regarded as the focused signal and was analyzed in the following. Figure 9 and Figure 10 present the received signal in the first step and the focused signal in the last step, respectively.

**Figure 11:**
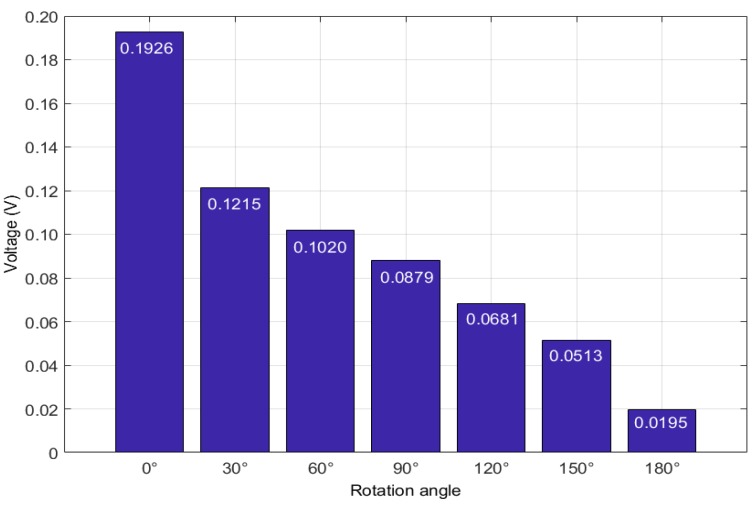
The detection result using the peak value of the focused signal.

From the figures it is clear that with the development of the connection looseness, which performed as the increase of the rotation angle θ, the magnitude of the received signal and focused signal all have a significate decrease. Following the theoretical description in Section 2, the peak value of the focused signal was selected as the loosening monitoring index to detect and monitor the looseness occurrence and development. As shown in Figure 11, the final detection result more clearly reveal the change trend that the monitoring index, i.e., the peak value, decreases with the development of the looseness. All the detection results demonstrate that the developed active sensing method combining with time reversal technique and piezoceramic transducers can effectively detect and monitor the looseness occurrence and development for the structures of threaded pipe connection.

### 4.2. Repeatability Verification

Repeatability of the developed method and consistency of the detection results are important for the structural health monitoring techniques especially in their practical applications. Therefore, an eight-repetition test was investigated in this research. The eight-repetition test was conducted in the same laboratory environmental condition, thus the environmental factors, including the temperature, moisture and pressure, were all considered constant and did not affect the test results. A discussion for the influence of the environmental factors can be found in Section 4.4.

**Figure 12:**
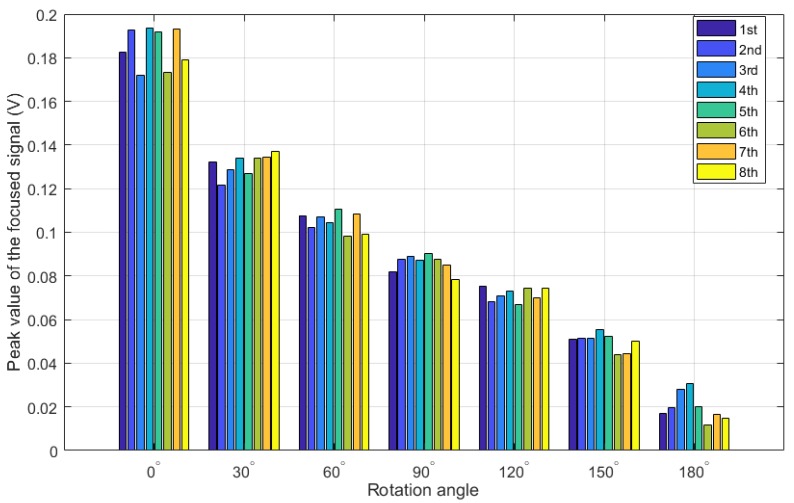
The peak value of the focused signal for the eight-repetition test.

Prior to each test in the experiment, the short pipe segment was firstly rotated to the initial tightened location, where the relative rotation angle was zero and the PZT actuator and sensor were located in a straight line along the length direction of the pipeline. For each test, the pipe part was then artificially rotated following the anticlockwise direction with an interval increase of 30° (1/12 circle) from 0 to 180° (1/2 circle) for the rotation angle. For the repetition test, a time interval with about 2 min was conducted between two consecutive measurements to save and pre-process the measured data. Figure 12 and Table 3 present the detection results for the eight-repetition test. Coefficient of variance (COV), which is the ratio of the standard deviation σ and mean value μ, was calculated and employed here to check the dispersion severity of the detection results. Finally, the repetition test results shown in the Figure and Table demonstrate that the developed loosening monitoring method combining with time reversal technique and piezoceramic transducers offers a good repeatability and consistency of monitoring the loosening status for the threaded pipe connection.

On the other hand, it should be noted from the Table that the COVs of the detection results under different loosening statuses with the rotation angle from 0 to 120° (1/3 circle) are all almost less than 5%, which further verified the good repeatability and consistency of the developed approach. However, with the looseness develops, the COV is then significantly increase to 7.85% at the rotation angle of 150° (5/12 circle) and 32.98% at the rotation angle of 180° (1/2 circle), which present much more sever dispersion than the ones under other initial tightening statuses. One reason for this phenomenon may be associated with relationship of the contact area and the rotation angle. With the development of looseness, i.e., the increase of the rotation angle, the helical clearance of the tapered thread between the coupling part and the pipe part becomes bigger and bigger, as shown in Figure 1a. Existence and development of the clearance allows a slightly shake for the pipe segment during its artificially rotating process in the eight-repetition test, and then induced a more serious randomness and uncertainty for the interface contact between the coupling and pipe part. Finally, these random contact interfaces were directly performed as the dispersion for the changes of the contact area. Therefore, it is speculated that when the looseness of the threaded connection develops to a certain degree, the interface contact area becomes more sever random and disperse, thus the loosening detection using the developed approach may be negatively effected in this situation. Based on the above analysis, it is concluded that the developed loosening detection method is more sensitive and suitable for the initial looseness condition.

### 4.3. Anti-Disturbance Ability

**Figure 13:**
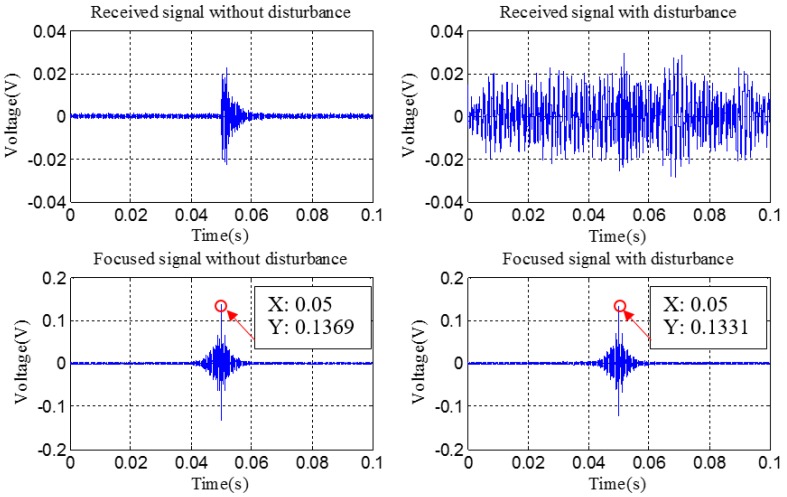
Anti-disturbance ability test with a loosening rotation angle of 30°.

In the practical application, a strong robustness ability for the noise and disturbance is very necessary and meaningful for the structural health monitoring method. In this study, the anti-disturbance ability was also investigated. The disturbances were artificially introduced by continuously rubbing the outside surface of the pipe part with a hammer. Then, two tests with the complete detection procedure using the developed method were successively conducted with and without the artificial disturbance, respectively. The received signals and focused signals under these two conditions were presented in Figure 13, in which, only the detection results under the rotation angle of 30° were presented because of the space limitation. From the figure it is observed that the received signal has been completely submerged by the artificial disturbance. Meanwhile, the peak values with and without the disturbance are almost the same. 

**Figure 14:**
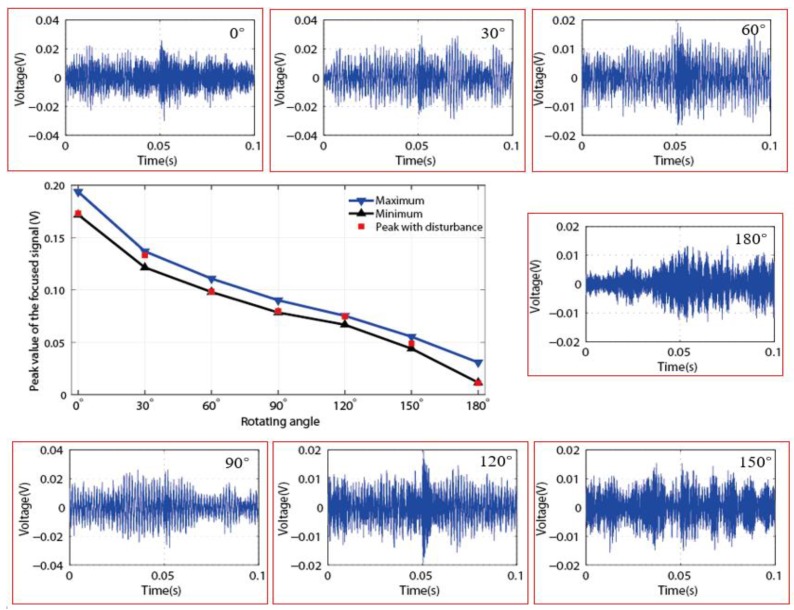
The anti-disturbance ability test for each loosening severities.

In order to further study the anti-disturbance ability of the developed method, all the seven loosening statuses represented by seven different rotation angles corresponding to different loosening severities, were employed and investigated using the developed method with the artificial disturbance, and the final results were presented as shown in Figure 14. The seven figures in the red frame are the received signals under seven test statuses with the environment of disturbance, and it is observed that all the received signals are completely submerged by the artificial disturbance when comparing with the ones under no disturbance conditions, which had been presented in Figure 9. In this situation, the peak values of the focused signals for seven different statuses with the disturbance were selected and plotted in Figure 14. In the figure, the maximum and minimum of the peak values collected from the eight-repetition test in Section 4.2 were also presented to indicate the allowance range for the monitoring indexes. Therefore, it is found that all the peak values collected under the disturbance environment are located in the allowance range. Based on these results, it demonstrates that the proposed monitoring index has a strong anti-disturbance ability to detect and monitor the looseness of the threaded pipe connection.

### 4.4. Discussion

By combining with time reversal technique and piezoceramic transducers, an active sensing method was developed in this study to detect and monitor the looseness occurrence and development of the threaded pipe connection by analyzing the stress wave propagation and attenuation between two PZT patches, and the effectiveness of the developed method had been validated by the experimental investigation of a threaded pipe specimen in laboratory. However, it should be noted that the lamb wave propagation and the final detection results of the developed piezoceramic-based method will also be influenced by some other factors, including environmental temperature, moisture, air pressure, test structural materials, the sensor style and location, etc. At the current stage, the study is focused on the feasibility of using the piezoceramic-based time reversal technique to monitor the occurrence and development of the connection looseness. Implementation of the developed method to monitor the looseness of real threaded pipe connection remains a future topic to study.

## 5. Conclusions

In this paper, a piezoceramic-based time reversal technique was developed to detect and monitor the looseness condition of the threaded pipe connection, and a laboratory specimen was prepared to investigate the effectiveness of the developed method. Two PZT patches were surface-bonded on the pipe part and the coupling part, respectively. The peak value of the focused signal can be utilized as an index to quantitatively estimate the looseness condition of the threaded pipe connection. Finally, the experimental results demonstrate that the peak value of the focus signal presents a significant decrease with the increase of the rotation angle of the pipe part, thus the looseness condition can be successfully monitored. In addition to the verification of the feasibility, the repeatability, consistence, and the anti-disturbance ability of the proposed method were also experimentally investigated. All the results demonstrate that the developed method has a great potential to monitor the looseness occurrence and development of the real threaded pipe connection, and offers a good repeatability and strong anti-disturbance ability.

## Figures and Tables

**Table 1:** The parameters of the specimen and the PZT patch.

Components	Parameters	Values	Unit
Steel specimen	Diameter (pipe part)	∅48 (∅42) ^1^	mm
	Diameter (coupling part)	∅60 (∅45) ^1^	mm
	Density	7900	kg/m^3^
	Young’s modulus	206	Gpa
	Poisson’s ratio	0.3	--
	Static friction coefficient (steel-steel)	0.15	--
PZT-5H	Dimension	∅12×0.5	mm
	Density	7800	kg/m^3^
	Young’s modulus	46	Gpa
	Poisson’s ratio	0.3	--
	Structural damping	3 × 10^−9^	--
	Dielectric loss factor	0.02	--
	Mechanical loss factor	0.001	--
	Piezoelectric strain coefficients *d*_31_, *d*_32_/*d*_33_/*d*_24_, *d*_15_	−2.10/5.00/5.80	10^−10^ m/V or 10^−10^ C/N
	Electric permittivity $\varepsilon_{11}^{T}$, $\varepsilon_{22}^{T}$/$\varepsilon_{33}^{T}$	1.75/2.12	10^−8^ F/m

^1^ the value in the bracket donates the inner diameter.

**Table 2:** Parameters of the pulse waveform.

Parameters	Value	Unit
Amplitude	10	V
Center frequency	200	kHz
Normalized bandwidth	0.02	--
Attenuation	2	dB
Delay	0.05	s

**Table 3:** Eight-repetition test results.

Rotation Angle	Min (V)	Max (V)	*μ* (V)	COV/*σ*/*μ*
0°	0.1719	0.1937	0.1847	0.0502
30°	0.1215	0.1369	0.1311	0.0386
60°	0.0980	0.1108	0.1048	0.0440
90°	0.0785	0.0901	0.0859	0.0459
120°	0.0668	0.0753	0.0716	0.0438
150°	0.0439	0.0553	0.0500	0.0785
180°	0.0115	0.0308	0.0197	0.3298
